# Case Report: Imaging findings of neonatal horseshoe lung with multiple system malformations

**DOI:** 10.3389/fped.2026.1849880

**Published:** 2026-06-25

**Authors:** Weixiu Wang, Haiyan Sun, Yalan Wu, Rongpin Li

**Affiliations:** Department of Radiology, Hebei Children's Hospital, Hebei Provincial Clinical Research Center for Child Health and Disease, Shijiazhuang, China

**Keywords:** computed tomography angiography, congenital pulmonary malformation, horseshoe lung, scimitar syndrome, VACTERL syndrome

## Abstract

This report describes the imaging findings of a neonatal case of horseshoe lung (HL) associated with multisystem malformations. The patient was a premature female neonate who presented with respiratory distress after birth. Physical examination revealed anal atresia with a rectovestibular fistula. Imaging examinations revealed multisystem malformations, including HL with abnormal bronchial branching and pulmonary hypoplasia, scimitar syndrome (infantile type), complex cardiovascular malformations, duodenal obstruction, and multiple vertebral anomalies. The application of multiple imaging modalities enabled comprehensive evaluation of the patient's condition and provided a basis for subsequent treatment and prognosis assessment.

## Introduction

HL is an extremely rare congenital pulmonary malformation characterized by fusion of the bilateral lower lung tissues across the midline posterior to the pericardium, forming a horseshoe-shaped structure. The term “horseshoe lung” was first coined by Spencer in 1962 to describe this anomaly (1). Fewer than 100 cases of HL have been reported in the literature to date, with most cases associated with scimitar syndrome, whereas association with VACTERL-spectrum anomalies has only rarely been reported (2, 3). The coexistence of HL and multisystem malformations poses distinctive challenges for clinical diagnosis and management. Herein, we report a neonatal case of HL complicated with scimitar syndrome, VACTERL association, annular pancreas and intestinal malrotation, aiming to enhance the comprehensive understanding of HL and its associated malformations.

## Case description

A female neonate, born via Cesarean section at 33 weeks, 2 days of gestation due to polyhydramnios and fetal distress, presented. At birth, she weighed 2.14 kg. Respiratory distress developed after birth, and because of prematurity, she was treated with endotracheal intubation, mechanical ventilatory support, porcine pulmonary surfactant replacement therapy, and anti-infective therapy with ceftazidime combined with penicillin.

Family history was not contributory, especially for congenital malformations. She is the second child in the family. Her mother had hypertension during pregnancy (140/100 mmHg) and was treated with oral labetalol.

Prenatal ultrasound suggested duodenal stenosis or atresia.

Physical examination after birth revealed congenital anal atresia with a rectovestibular fistula.

Cardiac and abdominal ultrasonography were performed on the 2nd day after birth. Echocardiography demonstrated patent ductus arteriosus (PDA) with an internal diameter of approximately 4.0 mm, dilatation of the main pulmonary artery, pulmonary hypertension (76 mmHg), patent foramen ovale, hypoplasia of the right pulmonary artery with an internal diameter of approximately 2.9 mm, poorly visualized left pulmonary artery, aberrant right subclavian artery, enlargement of the right heart, and moderate-to-severe tricuspid regurgitation. Abdominal ultrasonography revealed abnormal distribution of the intestinal loops within the abdominal cavity, with the colon located in the left abdomen and the small intestine in the right abdomen. An abnormal relationship between the superior mesenteric artery and vein was also noted, suggesting intestinal malrotation.

On the third day after birth, thoracoabdominal x-ray revealed a scimitar-shaped high-density shadow in the right middle and lower lung fields (scimitar vein). The stomach and duodenal bulb were dilated, presenting the “double bubble sign”, with no bowel gas observed in the mid-to-lower abdomen. Concurrently, multiple vertebral deformities were identified from T9 to T12, showing butterfly vertebrae at T9 and T11, as well as compensatory bulging of the inferior endplate of the T10 vertebra and the superior endplate of the T12 vertebra (Figure 1). Upper gastrointestinal series demonstrated obstructed passage of contrast medium through the descending duodenum, indicating a duodenal obstruction in the descending segment (Figure 2).

**Figure 1:**
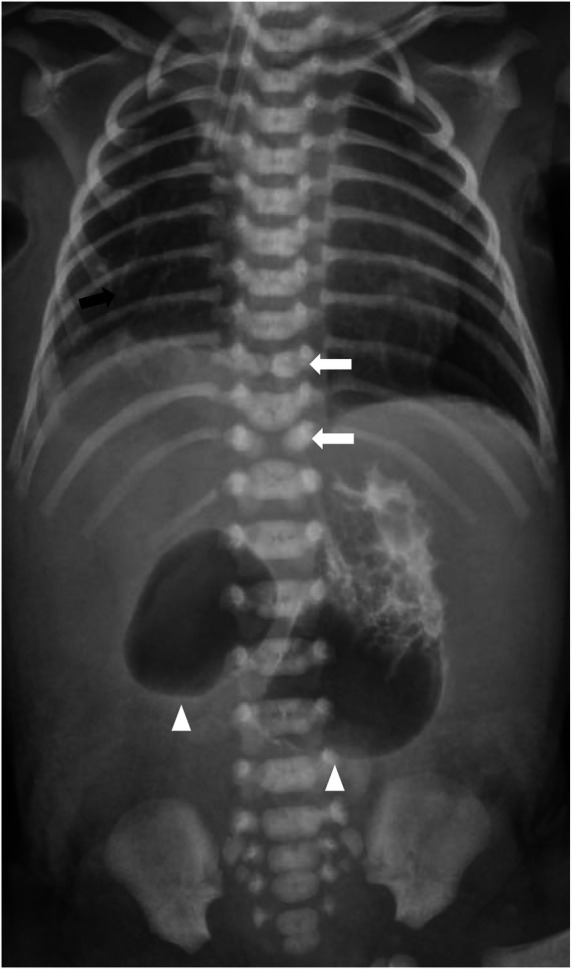
Thoracoabdominal x-ray. Scimitar vein (black arrow), and “double-bubble sign” in the upper abdomen (white arrowhead), and absence of bowel gas in the mid-to-lower abdomen. Multiple vertebral malformations involving T9-T12, butterfly vertebrae at T9 and T11 (solid white arrow).

**Figure 2:**
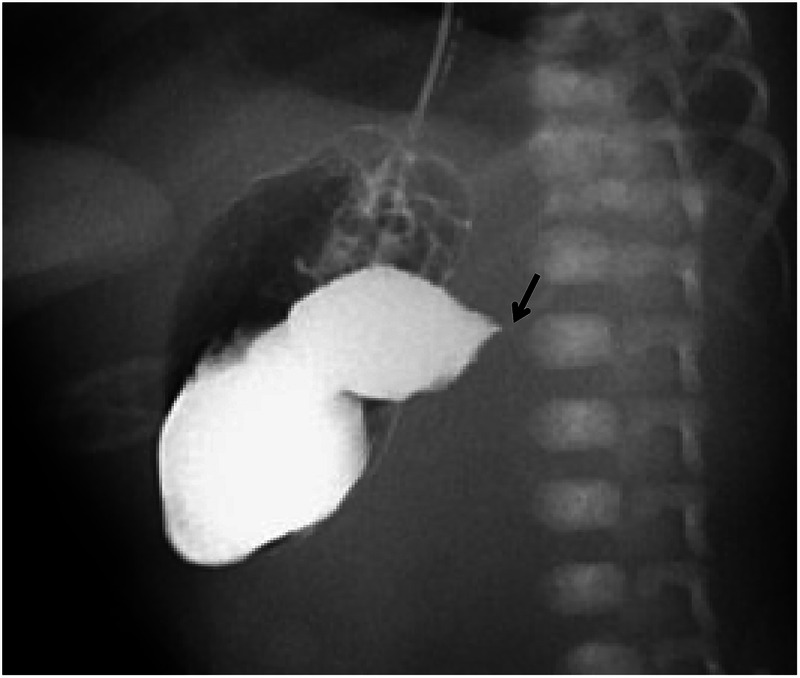
Upper gastrointestinal contrast study. Obstruction of the descending portion of the duodenum (black arrow).

Chest CTA performed on the fourth day after birth demonstrated fusion of the bilateral lower lungs through an isthmus of the lung parenchyma located anterior to the spine, thoracic aorta, and esophagus, and posterior to the heart. No significant difference in lung volumes was observed between the two lungs. No definite interlobar pleura were identified, and heterogeneous pulmonary lucency was present bilaterally. The trachea was deviated to the right, and the bilateral main bronchi lacked typical lobar bronchial branching patterns (Figure 3). Left-sided aortic arch with PDA. Coarctation of the aortic arch-descending portion, with the narrowest diameter measuring approximately 3.5 mm. The main pulmonary artery was dilated, with an internal diameter of approximately 9.0 mm. No left pulmonary artery was identified at the expected location; instead, a slender vessel was seen at the site of the right pulmonary artery coursing posteriorly and inferiorly. At the T4 level, this vessel bifurcated into the left and right pulmonary arteries. A branch of artery arising from the anterior aspect of the origin of the left pulmonary artery supplied the isthmus of lung parenchyma. The right pulmonary artery coursed rightward posterior to the left main bronchus and entered the right lung. The aberrant right subclavian artery originated from the aortic arch-descending portion and coursed posterior to the trachea and esophagus toward the upper right side. A large pulmonary vein from the left lung drained into the left atrium, while a curved pulmonary vein from the right lower lung drained into the inferior vena cava (Figures 4).

**Figure 3:**
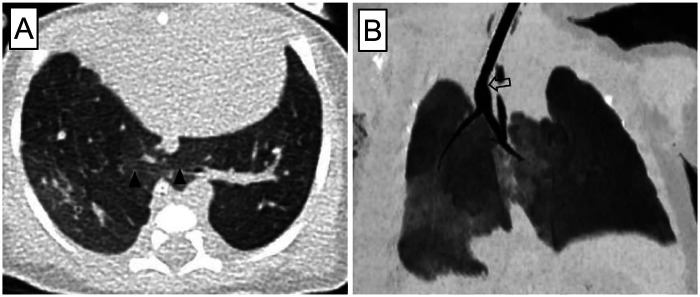
Chest CT scan. **(A)** The isthmus of lung parenchyma (black arrowhead). **(B)** The minimum intensity projection (MinIP). Rightward deviation of the trachea(open black arrow), with no obvious lobar bronchial branching from the left and right main bronchi.

**Figure 4:**
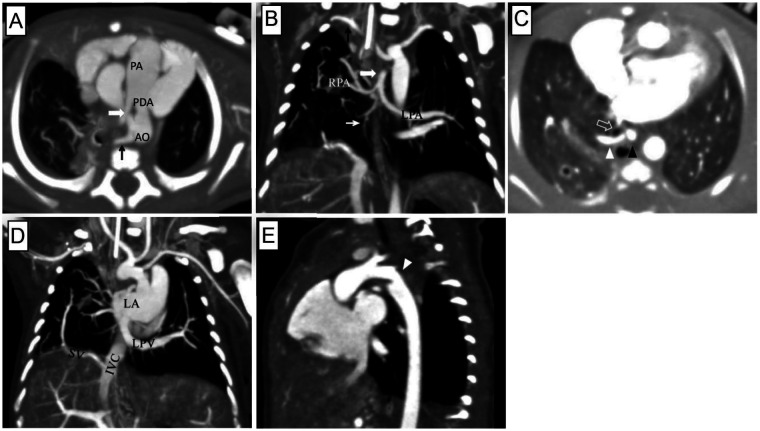
Maximum intensity projection (MIP) of thoracic vessels. **(A)** Left pulmonary artery is absent, while a slender vessel is seen at the expected location of the right pulmonary artery coursing posteriorly and inferiorly (solid white arrow). Aberrant right subclavian artery (black arrow). **(B)** The slender vessel (solid white arrow) bifurcated into the left and right pulmonary arteries. The arterial supply of the isthmus(white arrow). Aberrant right subclavian artery (black arrow). **(C)** The right pulmonary artery (white arrowhead) courses rightward posterior to the left main bronchus (open white arrow). The left pulmonary artery (black arrowhead). **(D)** A curved pulmonary vein from the right lower lung drained into the inferior vena cava, a large left pulmonary vein drains into the left atrium. **(E)** Coarctation of the aortic arch-descending portion (white arrowhead). AO, aorta; PA, pulmonary artery; PDA, patent ductus arteriosus; LPA, left pulmonary artery; RPA, right pulmonary artery. LPV, left pulmonary vein; LA, left atrium; SV, scimitar vein; IVC, inferior vena cava.

The patient was preliminarily diagnosed with: 1) prematurity and low birth weight; 2) neonatal respiratory distress syndrome and respiratory failure; 3) HL with abnormal bronchial branching and pulmonary hypoplasia; 4) scimitar syndrome (infantile type); 5) complex cardiovascular malformations, including PDA, aberrant right subclavian artery, and pulmonary arterial and venous developmental abnormalities; 6) persistent pulmonary hypertension; 7) obstruction of the descending portion of the duodenum, and intestinal malrotation; 8) congenital anal atresia with rectovestibular fistula; and 9) multiple vertebral malformations.

On the eighth day after birth, surgery was performed to relieve the upper gastrointestinal obstruction. Intraoperatively, a distinct transition zone between dilated and narrowed segments of the descending duodenum was observed, and annular pancreatic tissue encircling the duodenum was identified. An additional diagnosis of annular pancreas was therefore established.

On the 20th day after birth, the patient developed cough, fever, and respiratory distress, increased respiratory secretions were noted. Based on elevated C-reactive protein and white blood cell counts, together with pulmonary CT findings, neonatal pneumonia was diagnosed.

Whole-exome sequencing (WES) for molecular genetic analysis revealed no nucleotide sequence variants, copy number variants, or mitochondrial genome variants significantly or potentially associated with the clinical phenotype.

After 44 days of treatment, the patient tolerated enteral feeding through a gastric tube well and was able to defecate through the fistula. However, endotracheal intubation and mechanical ventilatory support were still required, and weaning from the ventilator remained unsuccessful. Following discussion, the family decided to discontinue treatment and discharge the patient against medical advice. Follow-up revealed that the patient died after discharge home.

## Discussion

HL is a rare congenital pulmonary developmental anomaly characterized by fusion of the bilateral lower lobes across the midline through an isthmus of lung parenchyma (3, 4). The isthmus is located posterior to the heart and anterior to the spine and thoracic aorta; in most reported cases, it is situated anterior to the esophagus (5, 6), whereas in a few cases it has been reported posterior to or even encircling the esophagus (3, 4). In the present case, the pulmonary isthmus was located anterior to the esophagus, consistent with most previous reports. No pleural structure was identified within the pulmonary isthmus, consistent with the features of true HL (4). As reported in the literature, angiography and bronchography revealed that the bronchial and arterial branches that supply the isthmus arise from the hypoplastic lung (6).

The embryologic origin of HL remains unclear, and two major hypotheses have been proposed. First, HL may result from a fusion process between lung parenchyma; second, the failure of visceral mesoderm to separate may lead to a connection between the lung and the pleural cavity (5, 6). Most reported cases lack a clearly defined genetic etiology (2). In the present case, molecular genetic testing revealed no abnormalities, which is consistent with previous reports.

HL is commonly associated with other complex pulmonary and cardiovascular malformations. The most frequent abnormalities are unilateral pulmonary hypoplasia (usually involving the right lung) and scimitar syndrome (4, 7). In the present case, the bilateral lungs were similar in size; however, both lungs lacked distinct interlobar fissures and lobar architecture, indicating bilateral pulmonary hypoplasia, a finding that has rarely been reported. The bilateral pulmonary arteries originated from a single arterial trunk arising posteriorly and inferiorly from the main pulmonary artery, and the right pulmonary artery coursed to the right posterior to the left main bronchus. This vascular variation has not been previously reported in the literature. Anatomically, the abnormal course of the pulmonary artery in this case shares certain similarities with a pulmonary artery sling; however, whether it represents a special subtype of pulmonary artery sling requires further investigation. It has been reported that approximately 80% of HL cases are associated with scimitar syndrome. Scimitar syndrome is characterised by a hypogenetic or hypoplastic lung with partial anomalous pulmonary venous drainage (4). The hypoplastic lung usually lacks normal fissures and lobes. The present patient also had complex cardiovascular malformations, including common abnormalities such as PDA, patent foramen ovale, and pulmonary arterial developmental anomalies, as well as less commonly reported abnormalities including aberrant right subclavian artery and localized aortic coarctation (3–6).

In addition to cardiopulmonary malformations, the present patient also had multiple vertebral anomalies and anal atresia, among other multisystem abnormalities, fulfilling the diagnostic criteria for VACTERL association. According to the literature, among 60 postnatally diagnosed cases of HL, 3 cases (5%) were associated with VACTERL association (3). HL has been reported in a limited number of patients with VACTERL association; however, current evidence is insufficient to establish HL as a component of the VACTERL spectrum. Other pulmonary anomalies including scimitar syndrome have been reported in association with the VACTERL spectrum. These associations may be a result of the same dysmorphogenetic process during blastogenesis (3). The association with oesophageal atresia, tracheo oesophageal fistula, congenital cystic adenomatoid malformation and preduodenal pancreas suggests that HL may be part of a foregut malformation (7, 8). This understanding may contribute to improving the comprehensive prenatal and postnatal diagnosis and management of VACTERL spectrum disorders.

The clinical manifestations of HL are variable. Patients may be asymptomatic or may present with recurrent respiratory tract infections, respiratory distress, cough, and symptoms related to associated malformations. Prognosis mainly depends on the severity of pulmonary hypoplasia and the extent of associated congenital anomalies (4, 7). The reported overall mortality rate is approximately 24% (2), highlighting the importance of identifying associated malformations. Comprehensive evaluation of related anomalies is essential for surgical planning.

HL should be differentiated from mediastinal pulmonary hernia. The absence of pleura within the isthmus of lung parenchyma and the presence of anomalous pulmonary venous drainage may suggest HL (6). Mediastinal pulmonary hernia results from pressure imbalance between the bilateral pleural cavities, with intact pleura visible between the two lungs, whereas pleural continuity is incomplete in HL (4).

This study is a single-case retrospective report; without autopsy findings. The relationship between horseshoe lung and VACTERL syndrome also requires further research to be confirmed.

## Conclusion

HL is a rare congenital anomaly that is commonly associated with complex cardiopulmonary malformations. HL is associated with scimitar syndrome, and may be associated with VACTERL spectrum disorders. Recognition of these associations should increase perinatal awareness of VACTERL spectrum disorders, thereby facilitating earlier prenatal or postnatal diagnosis and treatment. The application of multimodal imaging enables comprehensive and accurate evaluation of the patient's condition and provides an important basis for subsequent treatment and prognosis assessment.

## Data Availability

The raw data supporting the conclusions of this article will be made available by the authors, without undue reservation.
